# Evaluating Atlantic Salmon (*Salmo salar*) as a Natural or Alternative Host for Piscine Myocarditis Virus (PMCV) Infection

**DOI:** 10.3390/pathogens13090744

**Published:** 2024-08-30

**Authors:** Ingvild B. Nyman, Øystein Wessel, Håvard Bjørgen, Marta Alarcon, Torstein Tengs, Espen Rimstad

**Affiliations:** 1 Faculty of Veterinary Medicine, Norwegian University of Life Sciences, 1433 Ås, Norway; ingvild.nyman@nmbu.no (I.B.N.); oystein.wessel@nmbu.no (Ø.W.); havard.bjorgen@nmbu.no (H.B.); 2 Pharmaq Analytic, Harbitzaléen 2A, 0275 Oslo, Norway; marta.alarcon@zoetis.com; 3 Nofima, Osloveien 1, 1433 Ås, Norway; torstein.tengs@nofima.no

**Keywords:** piscine myocarditis virus (PMCV)

## Abstract

Cardiomyopathy syndrome (CMS) caused by piscine myocarditis virus (PMCV) has emerged with the rise of the aquaculture of Atlantic salmon (*Salmo salar*). The lack of cell culture cultivation has hampered the study of this infection. In this study, samples from naturally PMCV-infected Atlantic salmon from different commercial farms were collected and used. In situ hybridization (ISH) revealed intense staining of PMCV RNA in myocardial cells in the spongiform layer of the heart ventricle but almost no staining in the compact layer. In the kidneys, only sporadic staining was seen. Viral RNA was present in all organs, with the highest loads in the heart, kidney, and spleen. The high viral PMCV RNA loads in the heart were due to extensive viral mRNA transcription. The high ratio of viral mRNA to viral genomic dsRNA indicated active transcription but limited production of new viral particles. This suggests that the histopathological changes in the heart are caused by viral mRNA and corresponding viral proteins and not by virus particle formation. The production of full-length transcripts is regulated, with a reduction in the relative number of ORF3-containing transcripts at high transcription rates. Efforts to identify alternative hosts, such as fungi, were inconclusive, as fungal sequences were found inconsistently in the salmon tissue samples. The results of this study reinforce the need for further research to fully understand PMCV’s life cycle and potential alternative hosts and its whereabouts when it is not infecting the hearts of the Atlantic salmon.

## 1. Introduction

The development of the intensive large-scale marine aquaculture of Atlantic salmon (*Salmo salar*) has generated a melting pot for emerging infectious diseases. One example of a disease that has emerged in this industry is cardiomyopathy syndrome (CMS), a disease that predominantly appears in the later part of the salmon’s production cycle and has caused significant impacts on fish welfare and economic implications. CMS is commonly observed as a sudden increase in mortality in a net population, with or without the observation of preceding clinical signs. CMS typically emerges during the second year at sea, and with a relatively high number of outbreaks, it has a substantial economic impact on the Atlantic salmon production industry [1]. Diseased fish have normal to high condition factors; the gross pathological findings are circulatory disturbances due to acute or chronic heart failure, and the histopathology is characterized by subendocardial inflammation and myocarditis, degeneration, necrosis, and progressive fibrosis of the atrium and spongious myocardium [2]. The histological changes in CMS were described in the 1980s [3], but it took about twenty years until CMS was demonstrated to be a transmissible disease [4] and then subsequently found to be associated with infection with piscine myocarditis virus (PMCV) [5,6]. It has not been possible to cultivate PMCV in fish cell lines, and experimental infection is commonly performed by injecting each fish due to the slow horizontal transmission rate [7].

The physical structure of the PMCV particles resembles that of *Totiviridae* [5], with non-enveloped, single-layered, icosahedral particles composed of one major polypeptide. The genome of classical totiviruses consists of a single-molecule linear double-stranded RNA (dsRNA) with two open reading frames (ORFs): ORF1 encodes for the major capsid protein (MCP), and ORF2 codes for RNA-dependent RNA polymerase (RdRp). Due to ribosomal −1 frameshifting, ORF2 can be translated as a fusion with ORF1 approximately once every 100 translation events, causing some copies of the capsid proteins to be fusion proteins of MCP-RdRp [8]. Some totiviruses have an extra small toxin-encoding dsRNA in a satellite particle [9]. PMCV was found to be distinct from the classical totiviruses by having an extra ORF3 at the 3′-end of the genome, encoding a protein of unknown function [5].

Findings from both field and later experimental studies indicate that the horizontal transmission of PMCV from fish to fish may be very slow. For example, transmission of the infection from a heavily infected fish population in one net to another within a farm was observed to take several months, if occurring at all [2]. Similarly, it was observed that PMCV-infected fish used in a cohabitation challenge had transmitted the infection only to 54% of the naive fish in a tank after 24 weeks of cohabitation [7]; however, in earlier experiments, it was found that naive co-habitant fish in a tank became infected with a 4-week delay [5].

The totiviruses were originally associated with latent infections in unicellular organisms, such as yeast, protozoa, and fungi [10]. Viruses causing latent infections in unicellular organisms may never leave their host cell and do not need the capsid structures that recognize host cell receptors for the entry of cells from the extracellular environment, and the capsids thus primarily shield the genome from the cellular antiviral response and facilitate the synthesis of viral RNA. The transmission of the virus from one latent infected cell to another is dominantly vertical and occurs during cell division and cell–cell fusion [11]. By contrast, viruses with a capsid structure and a dsRNA genome, which enter cells from the external environment, must possess a mechanism for cell entry. For viruses tentatively listed as totiviruses infecting arthropods, such as the shrimp virus infectious myonecrosis virus (IMNV), it has been shown that the virus particles have fiber protrusions at the five-fold axes that are part of the machinery for extracellular transmission [12]. The totivirus Omono River virus (OmRV), which infects mosquitos, has an extra crown protein structure on the capsid that may facilitate horizontal cell-to-cell transmission [13]. The ORF1-encoded proteins of the arthropod-infecting totiviruses contain 2A-like motifs, which is an oligopeptide sequence mediating a ribosome skipping effect, producing two polyproteins without the need for an exogenous proteinase [14]. In IMNV, the 2A motifs give at least two polypeptides, in addition to the MCP, which are assumed to be part of the fiber protrusions and counteract the host cell’s antiviral response [15]. It can thus be argued that the evolution of arthropod-infecting totiviruses has led to the acquisition of fiber-coding sequences to allow extracellular transmission [12]. These viruses can be pathogenic to their hosts, and it has been shown that purified virions can reproduce the disease in pathogen-free animals [16]. Giardia lamblia virus (GLV), a totivirus able to enter its unicellular host from the extracellular environment, has surface loops, and similar ones are also present in the mosquito-infecting OmRV and are thought to be of importance for extracellular transmission [17].

Interestingly, the PMCV virion is in many ways similar to classical totiviruses by having ORF1 overlapping with ORF2; the size of the PMCV genome, 6.7 kbp, is within the range of the sizes of classical totiviruses, whereas the genome of the arthropod-infecting totiviruses is bigger (7.5 kbp) [15]. Also, there are no 2A-like motifs present in the ORF1 sequence of PMCV or in classical totiviruses, unlike in arthropod totiviruses [5], and there are no protrusions visible on the EM of the PMCV particle [5]. However, the presence of ORF3 in PMCV differentiates PMCV from the classical totiviruses.

In this study, we address the question of whether the Atlantic salmon is the natural host of PMCV. We characterized the infection dynamics and tissue tropism to determine whether they represent a natural infection and searched for indications of co-infection with other host organisms. Additionally, we investigated the genome transcription and organization of PMCV to see if it aligns with classical totiviruses.

## 2. Materials and Methods

### 2.1. Samples

All samples originated from commercial marine aquaculture sites for Atlantic salmon, where PMCV had previously been detected in the fish populations. These production sites were located along the Norwegian coast. Sampling was conducted by local fish health veterinarians who regularly monitored the health status of the fish populations at these sites. Typically, ten fish were included in each sampling, and the sampling procedure followed the national regulations outlined in “The Aquaculture Act” for animal welfare (“Regulation on the operation of aquaculture facilities §34: Killing of fish”).

At the time of sampling, the fish population was clinically assessed by the local fish health veterinarians as either apparently healthy or showing clinical signs of CMS. The fish were euthanized using a lethal dose of the anesthetic benzocaine chloride (1 g/5 L water). In one of the samplings, five of the ten sampled fish were dead at the time of sampling. Dissections were conducted aseptically, and the organs collected included the heart, head kidney, spleen, liver, gills, skin (from the base of the pectoral fin and skin scrapes), muscle, pyloric appendices, and mid- and hindgut. These samples were put in either RNAlater^®^ (LifeTechnologies, Carlsbad, CA, USA), L15 (Gibco, NY, USA), 96% ethanol, or PBS, and then shipped chilled overnight. Upon arrival, samples intended for RNA isolation were stored at −20 °C. Heart and kidney samples intended for in situ hybridization were fixed in 10% neutral buffered formalin, and after 24–36 h, the formalin was replaced with ethanol and the samples were stored at 4 °C.

Blood samples were centrifuged and the serum was collected and stored at −20 °C.

Additionally, heart and head-kidney samples from clinically healthy, PMCV-negative fish were sampled at the Centre for Fish Trials, Norwegian University of Life Sciences, Ås, Norway.

### 2.2. RNA Extraction

Total RNA was isolated using an RNeasy Mini QIAcube Kit (Qiagen, Hilden, Germany). Briefly, a 2 mm^3^ cube was sliced into small pieces and placed in a safe-lock tube with 500 µL buffer RLT (Qiagen) with 40 mM DTT and a 5 mm steel bead (Qiagen). The tissue was disrupted and homogenized in a TissueLyser II (Qiagen) for 5 min at 25 Hz. Total RNA was isolated according to the instructions of the manufacturer and eluted in 30 µL RNase-free water.

Viral RNA was isolated using the QIAamp Viral RNA Mini Kit (Qiagen). A 140 µL tissue homogenate was mixed with 560 µL buffer AVL (Qiagen) containing carrier RNA, and viral RNA was isolated following the manufacturer’s protocol and eluted in 50 µL buffer AVE (RNase-free water with 0.04% sodium azide).

### 2.3. Extraction of Virion dsRNA

A 1 mm^3^ cube was placed in a safe-lock tube with 250 µL buffer (10 mM Tris, 1 mM EDTA, pH 8.0) and a 5 mm steel bead (Qiagen). The tissue was disrupted and homogenized in a TissueLyser II (Qiagen) for 10 min at 25 Hz. After homogenization, 4 U PureLink^TM^ RNase A (LifeTechnologies) was added and the sample was incubated at 37 °C for 30 min. Thereafter 4 U DNase (ThermoFisher, Vilniaus, Lithuania) was added and the sample was further incubated at 37 °C for 30 min. Viral RNA was isolated as described above.

### 2.4. RT-qPCR and Sequence Analyses

For the detection and estimation of the amount of viral RNA, the Brilliant III Ultra-Fast QRT-PCR Master Mix (Agilent Technologies, Santa Clara, CA, USA) was used. The RT-qPCR was set up using the following conditions: 1× QRT-PCR Master Mix, 30 nM ROX reference dye, 400 nM primers (Table 1), 300 nM probe (Table 1), 1 mM DTT, 0,75 µL RT/RNase block, and 100 ng total RNA. The test was then carried out in a total volume of 15 µL.

Prior to the RT-qPCR testing, the RNA was denatured at 95 °C for 5 min, followed by a reverse-transcription phase for 10 min at 50 °C, a hot-start phase at 95 °C for 3 min, and 40 cycles of 95 °C for 5 s and 60 °C for 10 s. The samples were run in duplicate on AriaMx (Agilent Technologies, Penang, Malaysia), and were considered positive if both parallel samples had a Cq value < 40.

To test the efficiency and reproducibility of the PMCV primer sets, cDNA from the total RNA of heart samples from two fish were serially diluted in a ratio of 1:10 and used as templates.

Additionally, the National Center for Biotechnology Information (NCBI) Sequence Read Archive (SRA) was mined for datasets from CMS-infected material. These data were analyzed bioinformatically for the presence of non-salmonid sequences using an approach previously described by our group [20].

### 2.5. Detection of ssRNA and dsRNA—RACE

Total RNA (1 µg) isolated from the heart and head-kidney samples with a PMCV Cq value of 24 or lower was incubated with 1.12 U PureLink™ RNase A at either a low salt concentration or a high salt concentration (0.5 M NaCl). At 0.5 M NaCl, dsRNA is selectively protected from RNase A degradation, but ssRNA is not [21]. Following incubation with the RNase A at 25 °C for 45 min, RNA was recovered using an RNeasy MinElute Cleanup Kit (Qiagen) as per the manufacturer’s instructions.

We also adapted the method established by Polinski et al. for quantifying reoviral ssRNA and dsRNA [22]. The principle behind this method is that dsRNA segments will not be transcribed to cDNA unless they are first denatured into single strands by heating (95 °C for 3 min).

To characterize 5′ ends from potentially low-copy-number viral transcripts that do not correspond to full-length genome copies, we performed a Rapid Amplification of cDNA Ends using a 5′ RACE System following the manufacturer’s instructions (Thermo Fisher Scientific, Waltham, MA, USA).

### 2.6. Statistical Analysis

The amounts of PMCV dsRNA and ssRNA found with the RT-qPCR tests in different organs were compared using Student’s *t*-test.

### 2.7. In Situ Hybridization (ISH)

Chromogenic detection was performed using the RNAscope^®^ 2.5 HD Detection Kit RED (Advanced Cell Diagnostic (ACD), Newark, CA, USA) for detecting PMCV in tissue samples. According to the manufacturer’s instructions, paraffin-embedded tissue sections (5 µm) of hearts and kidneys from the PMCV-infected fish and control fish were dewaxed at 60 °C for 90 min in an ACD HybEZ™ II oven, followed by hydrogen peroxide treatment for 10 min at room temperature. The samples were then boiled in RNAscope target antigen retrieval reagent for 15 min and then incubated with RNAscope protease plus at 40 °C for 15 min in a HybEZ™ oven. Each section was hybridized with an RNAscope probe (Table 2) designed against the positive strand (mRNA) of PMCV-ORF1 or PMCV-ORF2 or the negative strand (genomic RNA) of PMCV-ORF2 (ACD; catalog numbers 812021-C2, 555231, 1219761-C3) for 2 h at room temperature. A probe targeting Peptidylpropyl Isomerase B (PPIB) in Atlantic salmon (ACD; catalog number 494421) was used as reference target gene to test for RNA integrity in the samples. As a negative control, probe-DapB (ACD; catalog number 310043) was used to evaluate cross reactivity. Fast Red chromogenic substrate was used for signal detection following the manufacturer’s instructions. The samples were counterstained with 50% Gill’s hematoxylin solution and mounted with EcoMount (BioCare Medical, Pacheco, CA, USA).

Imaging was performed via bright-field microscopy with a DM6B-Z microscope (Leica Microsystems, Wetzlar, Germany).

For fluorescent in situ hybridization (FISH), we used the RNAscope^®^ Multiplex Fluorescent Reagent Kit v2 (Advanced Cell Diagnostics). The samples were treated similarly to the RNAscope chromogenic method. The probes were assigned a fluorophore, Opal 520, with emission/excitation wavelengths of 494/525. Each section was counterstained by adding DAPI (a fluorescent DNA stain) for 30 sec at room temperature. Mounting was performed by adding 1–2 drops of Prolong Gold antifade mounting reagent (Thermo Fisher Scientific).

Fluorescent images were captured using a DMi8-CS confocal microscope (Leica Microsystems, Wetzlar, Germany) or the DM6 B Thunder microscope (Leica Microsystems, Wetzlar, Germany). Lasers with wavelengths of 405 (DAPI) and 488 (FITC) nm were used.

## 3. Results

### 3.1. In Situ Localization of PMCV RNA in Heart Ventricles and Kidneys

To determine the localization of PMCV RNA in the heart ventricle, ISH protocols targeting the PMCV-ORF2, which encodes the RNA-dependent RNA polymerase gene, were established. The specificity of the detection was validated by the use of positive and negative ISH controls, as well as by the fact that probes against PMCV ORF1 and ORF2, i.e., different parts of the PMCV genome, recognized the same cells (Figure S1).

**Figure 1:**
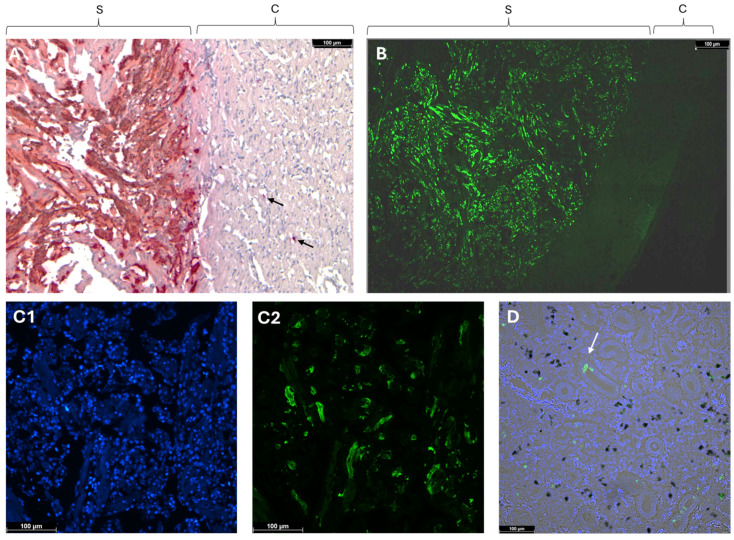
In situ hybridization of the heart for PMCV with (**A**) chromogenic staining (red) and (**B**–**D**) fluorescent staining (green). (**A**) Chromogenic staining. Staining is restricted to the stratum spongiosum with only a few stained spots (arrows) in the compactum. Cq = 13 for the sample used, indicating a high viral load. Lens: 10× objective. (**B**) Fluorescent in situ hybridization of a transverse section of the ventricle. Staining is restricted to the stratum spongiosum. Lens: 10× objective. (**C**) DAPI staining (**C1**)—where blue dots represent nucleated hematocytes, including red blood cells—and fluorescent in situ hybridization (**C2**) of the stratum spongiosum against PMCV. The same section is shown in (**C1**,**C2**), and no PMCV staining of the hematocytes is seen. Lens: 20× objective. (**D**) Scattered staining of the kidney for PMCV. Sample was counterstained with DAPI. Black spots represent melano-macrophages. Arrow points to staining in the tubular structures. S = spongiosum; C = compactum.

Chromogenic detection in heart samples with low Cq values, indicating high viral RNA loads, showed an intense and confluent staining of myocardial cells in the stratum spongiosum with a sharp demarcation to the stratum compactum (Figure 1A). Only a few positive cells were detected in the stratum compactum. The intense and confined staining in the spongious layer, with a sharp demarcation to the compact layer, was confirmed by fluorescent ISH probes. A cross-section of the heart ventricle displayed strong staining in the spongiform trabeculae with no signal being observed in the compact and epicardial layers (Figure 1B). This strong, well-defined staining in the spongiform layer of the heart observed with ISH aligns with previously described histopathological changes, which were largely restricted to the spongy portion of the heart [23]. Counterstaining for nuclear DNA revealed that PMCV infection was not observed in the blood cells filling the space between spongiform trabeculae (Figure 1(C1,C2)), and PMCV RNA was only detected in parts of the spongiosum trabeculae.

In contrast, ISH staining for PMCV in the kidney samples was sporadic and punctate (Figure 1D). There was no indication of staining of the kidney’s melano-macrophages; however, positive signals for PMCV were observed in the tubular structure (Figure 1D: arrow), possibly indicating shedding or infection of the kidney epithelial cells. The scattered presence of PMCV RNA in the kidney sharply contrasts with the extensive staining seen in the hearts of individuals with a high viral RNA load.

### 3.2. PMCV RNA Loads in Different Organs

Ten fish from Farm A were sampled two months after PMCV was detected at the farm. At the time of sampling, the fish did not show any clinical signs of disease and there was no increased mortality. Six of the fish tested positive for PMCV by RT-qPCR in all organs examined, with two of these fish showing high viral loads in the heart, indicated by Cq values of 16.4 and 16.9. In the PMCV-positive fish, the highest viral RNA loads were found in the heart, kidney, and spleen, with average RT-qPCR Cq values of 21.9, 23.1, and 23.2, respectively, and these levels were not significantly different. Significantly lower viral RNA loads were detected in the gills, liver, muscle, skin, pyloric ceca, and mid- and hindgut, with average Cq values ranging from 26.2 to 29.1 in positive fish (Table 3). One fish was positive only in its heart, kidney, and skin-scrape samples, while another was positive only in its skin scrape. Two fish were negative for PMCV in all of their samples when tested with RTqPCR (Table 3). As with field samples in general, the exact extent of the PMCV exposure among this population is unknown. The efficiency and reproducibility of the primer set and probes used here have been previously determined [6].

Further analyses of the viral RNA loads focused on the heart and kidney, representing two organs with high viral loads. Ten fish from Farm B and ten from Farm C—farms where, similarly to Farm A, the fish did not show any clinical signs of disease at sampling—had average Cq values in the heart and kidney of 19.5 and 22.6 (Farm B) and 21.0 and 20.3 (Farm C), with no significant differences between the organs.

In contrast, at Farm D, where the fish had tentative clinical signs of CMS at the time of sampling, the viral RNA load was significantly higher (*p* < 0.01) in the heart samples compared to the kidney samples, with average Cq values of 14.6 ± 1.8 and 20.4 ± 0.94, respectively.

### 3.3. PMCV RNA Forms in Various Organs at Low and High Viral Loads

The findings from the ISH and RT-qPCR analyses suggest that in some individual fish, PMCV replicates extensively in the spongiform part of the heart ventricle, but to a lesser extent in other organs such as the kidneys. To investigate whether this extensive replication in the heart results in a high production of new viral particles, potentially making fish with clinical symptoms particularly important in the spread of the disease, we sought to examine which form of viral RNA is present in different organs.

To determine if PMCV RNA is present as dsRNA genomic RNA (gRNA) or viral mRNA in infected cells, we hypothesized that PMCV gRNA would not be detected by RT-qPCR tests in infected cells unless it was denatured into single strands by heating prior to the reverse transcription of cDNA. This hypothesis was based on an established method designed to distinguish between the genomic dsRNA and ssRNA transcripts of the piscine orthoreovirus [22].

To validate the method, we used total RNA from two heart samples that showed no differences in the obtained Cq values based on whether the extracted RNA was heated at 95 °C for 5 min or not prior to cDNA synthesis. The results revealed that for such samples, a major part of the RNA is likely ssRNA. When RNA was digested with RNase A in the presence of 0.5 M NaCl, which selectively degrades ssRNA, a difference was found in the Cq values (ΔCq) relative to the values obtained when no RNase A digestion was used: for the two test samples, this difference was 15.4 and 16.8, corresponding to a reduction in the RNA target by approximately 1:65,000. RNase digestion in low-salt conditions completely degraded the RNA. This demonstrated that most of the RNA detected by RT-qPCR without denaturing prior to cDNA synthesis was indeed ssRNA.

To assess whether the relative proportion of PMCV gRNA versus mRNA differed between organs and between fish with or without clinical symptoms, we tested samples from the heart and kidney. In the heart, PMCV ssRNA made up close to 100% of the detectable total RNA for 9 out of the 10 fish selected for a low Cq in the heart, with an average ΔCq of 0.02 between the heated and non-heated samples (Table 4).

In contrast, the findings in the kidney were remarkably different, with an average ΔCq of 3.4. In the kidney, the ssRNA made up only 9.4% of the total viral RNA, while the genomic dsRNA made up more than 90% of the viral RNA. Even in individuals where nearly 100% of the detectable viral RNA in the heart was ssRNA, the dominant form in the kidney was genomic dsRNA (Table 4). This indicates that during the acute phase of infection, characterized by high viral RNA loads, the primary viral process in the heart is viral mRNA transcription, with no detectable synthesis of dsRNA that would indicate particle formation. In the kidney, however, the dsRNA was the dominating form, and the extensive mRNA production found in the heart was not detected.

### 3.4. Genomic Organization and Transcription of PMCV

**Figure 2:**
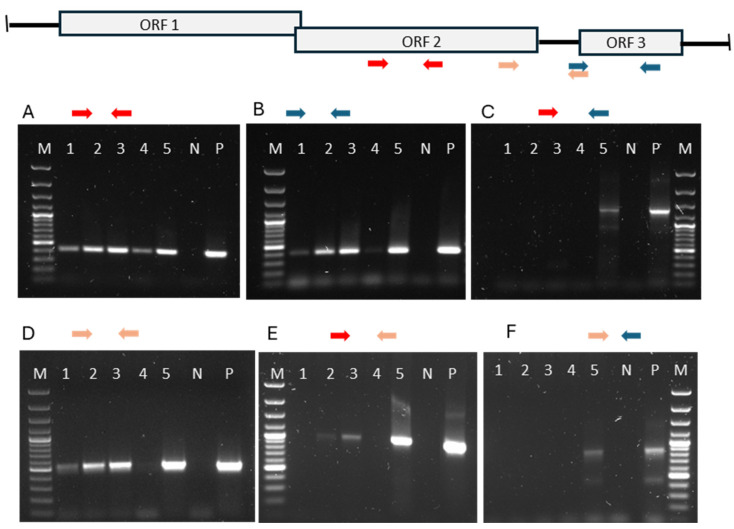
Upper panel: Locations of primers used in the RT-qPCR testing of extracted viral genomic RNA from intact virus particles. Samples were treated with RNase prior to RNA extraction. The color-coded arrows indicate the positions of primers from ORF2 and ORF3. Middle and lower panels (**A**–**F**): The different combinations of primers used. The color-coded primers are shown at the top of each gel. M = molecular marker; P = positive control, i.e., total RNA extracted from non-RNase-treated material; N = negative control.

The detection of viral RNA by RT-qPCR using extracted, heat-denatured total RNA does not differentiate between viral genomic RNA (gRNA) and transcribed ssRNAs. Genomic viral RNA within intact viral particles is protected against nucleases. Thus, to specifically detect viral gRNA, cDNA synthesis must be preceded by RNase treatment, which degrades viral mRNA outside viral particles but leaves the RNA genomes within intact viral particles undamaged. This method is used to distinguish between infectious and non-infectious viral particles, assuming that intact particles are infectious [24]. In our study of viral genomic RNA versus mRNA, we used samples that were stored in PBS, rather than in an RNase-inhibiting fixative. These samples were treated with RNase A prior to RNA extraction. The extracted viral genomic RNA was then used in RT-qPCR tests with primers targeting ORF2 or ORF3. Results from the RNase treatment prior to RNA extraction and RT-qPCR are shown in Figure 2. The RT-qPCR tests with primers from either ORF2 or ORF3 both gave a correctly sized product (264 bp and 266 bp), demonstrating that the preceding RNase treatment had not destroyed the genomic RNA (Figure 2A,B). When combining primers from ORF2 and ORF3, i.e., primers shown as red and blue arrows, no amplification was seen, apart from in one sample (Figure 2C), which could indicate a discontinuous genome, as seen in toxin-producing totiviruses [9]. However, without preceding RNase treatment, where full-length mRNA transcripts were present, amplification was achieved with the ORF2 + ORF3 primers, as seen in lane P in Figure 2C, suggesting a continuous genome. A Rapid Amplification of cDNA Ends (RACE) procedure was carried out but it did not provide evidence for discontinuity. Figure 2D–F show that amplification of parts of ORF3 is only partly successful when using viral genomic RNA as the target (Figure 2C,F) but is more successful when total RNA (lane P), which also contains viral mRNA, is included as a target.

Totiviruses produce a full-length copy of the genome which is extruded from the cytoplasmic particle [25]. The difficulty we encountered in amplifying parts of ORF3 from genomic RNA could suggest structural issues for the reverse transcriptase used in the cDNA synthesis of the RT-qPCR. Alternatively, it may indicate a lower number of mRNA transcripts containing ORF3 compared to those containing ORF1/2. We first assessed the efficiency and reproducibility (R^2^) of different primer sets, which ideally should be between 90 and 110% and higher than 0.980, respectively. The primer sets of ORF2, SP3-orf2Fw-orf2Rw, and of ORF3, orf3Fw-SP3-orf3Rw (Table 1), showed efficiencies of 100% and 93%, respectively, with both having an R^2^ of 0.99. The sizes of these amplicons were 264 bp and 266 bp, respectively. We used total RNA from heart and kidney samples of four fish that had not undergone preceding RNase treatment or heat denaturing before cDNA synthesis. Thus, the RT-qPCR tests mainly detected ssRNA. The relative abundance of ORF2-containing PMCV transcripts in the heart relative to that in the kidney was approximately 1000:1, as indicated by the ΔCq value between the heart and kidney being −10, and 2^10^ equals 1024 (Table 5). The proportion of amplicons from ORF3 ssRNA versus ORF2 ssRNA was between 3 and 10% in the heart samples and between 19 and 40% in the kidney samples (Table 5). While differences in primer sensitivity could explain some variation, they do not explain the differences between the organs. The relative abundance of ORF3 versus ORF2 transcripts between the heart and kidney was statistically significant (*p* = 0.0020). In the heart, ssRNA containing ORF2 was, on average, sixteen times more prevalent than ORF3, and in the kidney, it was about three times more prevalent.

To verify this with different primer/probe sets, we used the PMCV-F/R/probe (ORF2) and ORF3 F/R/probe (ORF3) (Table 1) on 15 heart samples. The obtained average Cq values were 19.6 for ORF2 and 24.3 for ORF3. The ΔCq was 4.7 between ORF2 and ORF3, indicating that there were 26 times more ORF2 transcripts than ORF3 transcripts.

Overall, these results suggest that transcripts containing ORF3 are less abundant than ORF2-containing transcripts, particularly in the heart at high rates of transcription activity.

### 3.5. Search for Alternative Host Organisms for PMCV Present in Atlantic Salmon

**Figure 3:**
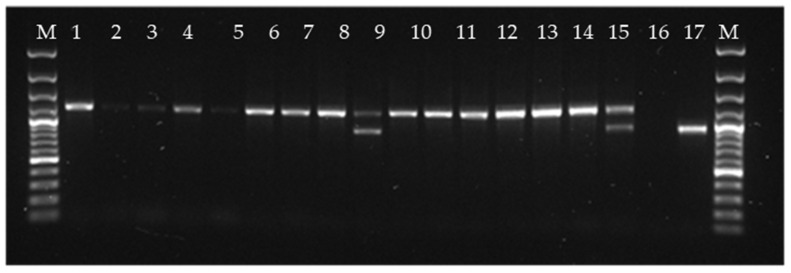
PCR amplification using primers developed for the detection of conserved parts of ribosomal DNA of the fungus Malassezia. The large majority of sequences corresponded to ribosomal DNA of Atlantic salmon, while the sequences of the bands in lanes 9, 15, and 17 matched Malassezia sp.

The suggestion that the expression of viral transcripts containing ORF3 is regulated may imply that ORF3 could function similarly to the toxin gene found in totiviruses of unicellular organisms, wherein the toxin gene is found in a satellite particle. Although we did not find evidence of a discontinuous genome for PMCV, the characteristic changes in the atrium and spongious myocardium associated with CMS could indicate a toxic effect. Moreover, several studies have reported that PMCV does not spread efficiently from fish to fish [2,7], suggesting that the capsid stability could be a limiting factor for virion survival in the external environment. This could warrant investigations into potential host organisms other than salmon. To explore this possibility, we used PCR tests with primers designed for the amplification, sequencing, and subsequent barcoding of conserved genomic regions across various genera. These primers are listed in Table S1 (Supplementary Materials). DNA was extracted from heart and kidney samples of salmon that tested positive for PMCV via RT-qPCR. The results were negative for all primer pairs listed in Table S1, except for the LROR/LR5 pair, which detected the large-subunit rRNA of fungus in some individual fish (Figure 3). The sequence of the amplicon of approximately 1.2 kb in size, seen in almost all lanes in Figure 3, matched that of *Salmo salar* ribosomal DNA. However, the sequence of the amplicon of approximately 1.0 kb in size, seen in lanes 9, 16, and 18, matched that of *Malassezia restricta* and *Debaryomyces* sp. with 99.9% identity. Malassezia is a known host for totivirus [26] and Debaryomyces is a genus of yeasts in the family Saccharomycetaceae, also a known host of totiviruses [27]. This finding suggests that Malassezia or Debaryomyces might be present in some PMCV-positive samples. However, this raises the question about whether this is an accidental findings or an indication of contamination during the sampling process. If these organisms were mandatory hosts for PMCV, one would expect all PMCV-positive samples to be PCR-positive for these agents. To determine if this finding was accidental, two approaches were undertaken. First, PCR testing was performed with primers specific for Malassezia and Debaryomyces (Table S1), which yielded only negative results, suggesting that the initial finding was coincidental. Additionally, we analyzed related fungal species in other Next-Generation Sequencing (NGS) datasets generated from PMCV-positive material. A relevant dataset was identified in the NCBI’s SRA database, originating from a 2010 study [6] (accession no. SRP002117) on the experimental transmission of CMS that revealed numerous reads matching *Malassezia restricta*. This was the only fungal species that could be identified using our bioinformatics pipeline.

## 4. Discussion

The strong correlations found between viral RNA loads, the location of the viral RNA, and the severity of histopathological changes are robust indicators of PMCV’s role in the etiology of CMS [5]. However, the challenges in propagating PMCV in cell lines has made controlled experimental infections difficult, and no studies have been published to date that reproduce CMS in fish using purified virus particles. Although replication of PMCV has previously been indicated in both CHH-1 and GF-1 cells [5], these observations have been difficult to replicate. Consequently, experimental infection protocols for PMCV typically use injections of material from fish that have tested positive for PMCV by RT-qPCR [7,28], meaning that, experimentally, PMCV-infected fish are not infected through the virus’s natural route. In the current study, samples were collected from naturally PMCV-infected fish in commercial farms. The nature of field sampling implies uncertainty about the timing of the introduction of the virus to the fish population, and, furthermore, as horizontal transmission PMCV is observed as a slow process [2,7], one would expect significant individual variation in the progression of infection within a naturally infected fish population.

We tested fish from farms where the populations had previously tested positive for PMCV by RT-qPCR. At the time of sampling, the fish predominantly showed no clinical or pathological signs of circulatory disturbance. In most fish, PMCV RNA was found in all organs tested. For PMCV-infected fish without clinical symptoms, the highest viral RNA loads were found in the heart, kidney, and spleen, with a relatively even distribution of viral RNA among these organs, as indicated by the Cq values. However, some individual fish without clinical signs had higher viral RNA loads in the heart compared to the kidney and spleen, with Cq values of approximately 16 versus 22–23.

In fish that were assessed as having clinical signs consistent with CMS at the time of sampling, and therefore were assumed to represent the clinical phase of the disease, a significantly higher amount (*p* < 0.01) of viral RNA was detected in heart samples compared to kidney samples. This corresponds well with previous research indicating that the heart is the prime target organ for this virus during the acute phase [5]. Furthermore, the RT-qPCR results were consistent with the ISH findings and earlier findings where PMCV was found to be almost exclusively present in the lesions when micro-dissecting inflamed ventricular tissue [29]. Interestingly, PMCV was detected in skin-scrape samples, i.e., mucus, in some individuals that had very low levels or no detectable PMCV in their heart and kidney samples, which could suggest transmission through water.

A natural follow-up step was to study the viral transcription activity. During the process of naked, icosahedral viruses with dsRNA genomes entering cells from the exterior, i.e., crossing the cellular membrane, they lose parts of their capsid structure and reorganize to form transcriptionally active core particles that are released into the cytoplasm. These core particles synthesize full-length, positive-sense, non-polyadenylated single-stranded mRNAs (ssRNAs) [27,30]. The viral mRNAs are identical to the positive strand of the dsRNA genome and are either translated or packaged into new particles before the synthesis of the complementary strand. Thus, two types of viral RNA exist within the cell: dsRNA, which represents viral genomic RNA (gRNA), and ssRNA, which is synthesized and either capped to act as mRNA for viral protein translation or incorporated into new assembling particles, serving as a template for the viral polymerase in the minus-strand synthesis. The relative amount of viral single-stranded RNA transcripts is therefore an indicator of active transcription, while double-stranded genomic RNA indicates the presence of old or newly made viral particles. We validated that absence of RNA denaturing prior to cDNA synthesis could be used to distinguish between totiviral double-stranded genomic RNA and single-stranded RNA [22], by comparing heat-denatured samples to samples treated with the enzyme-based method of RNase A treatment using high or low salt concentrations.

It is natural that the ratio of PMCV gRNA to mRNA in infected cells varies through the infection process. A very high ratio of viral mRNA compared to gRNA was observed in hearts with a high viral RNA load, and in some fish, the only viral RNA detected in the heart was mRNA, i.e., the gRNA was below the detection limit of the method used. In fish with a low viral RNA level in the heart, the ratio between viral mRNA and gRNA was much lower. The relative amount of viral mRNA versus gRNA in individuals regarded as clinically affected with CMS indicated a massive transcription activity. In an earlier study, Cq values for PMCV in the heart were found to be as low as 7 [2]. However, we did not find indications that the massive viral transcription in the heart was reflected in the production of new viral particles as the amount of dsRNA was low, indicating either a lack of packaging of the positive-strand transcripts or a lack of the subsequent synthesis of the negative strand. Both the chromogenic and fluorescent ISH clearly demonstrated that the presence of a large amount of viral RNA was restricted to the spongiform part of the heart ventricle, with very little staining found in the stratum compactum. There is no connective tissue layer between these two muscle compartments of the heart ventricle [31]. Together, this indicates that in fish with high loads of viral RNA in the heart, typically those showing clinical signs of CMS, the high viral RNA loads are due to viral transcription that is restricted to specific parts of the heart. We did not observe any spreading of the virus from the spongiform layer to nearby heart compartments. The massive viral transcription in the heart was not present in the kidney, and consistent with this, there was only punctual ISH staining found in this organ.

The pathological effects observed in the spongiform part of the heart are due to the presence of viral RNA and proteins and not the production of new virus particles. Degeneration and necrosis of the inner, spongious myocardium of the ventricle are characteristic histological findings. However, infiltration with inflammatory cells and proliferation of the endocardium are also observed in CMS, which indicate an immune response and recovery phase [23,32]. The ORF3 protein is highly cytotoxic and harmful to cells and is considered important for the pathogenesis of CMS [33]. Toxin production is well described for totiviruses of yeast [34], where the toxin is encoded by a dsRNA segment that is encapsidated in a satellite capsid. The segmentation of the genome is increasingly being found in totiviruses of various hosts, such as fungi [26]. This encouraged us to study the organization of the genomic RNA of PMCV. The genome of naked, icosahedral virus particles is protected against RNase activity in intact viral particles, while viral RNA outside the virion is not similarly protected [24]. Therefore, we collected field samples in PBS, i.e., without any RNase inhibitor in the sample collection medium, treated these samples with RNase, and extracted the RNA. RT-qPCR tests were run separately for ORF2 and ORF3 and we obtained the expected amplicons, which demonstrated the presence of viral genomic RNA after the RNase treatment. When we ran an RT-PCR of this material with primers from ORF2 together with ORF3 primers, we did not obtain amplification for most samples, which could indicate that there was discontinuity in the genomic dsRNA. However, when the preceding RNase treatment was omitted, and thus the full-length transcripts were present, a product of expected size was amplified using the ORF2 + ORF3 primers. This finding did not indicate a discontinuation of the genome. Previous sequencing of PMCV and other totiviruses found in fish has not indicated segmented genomes [5,35]. Our RT-qPCR results using primer pair combinations from various regions of ORF2 and ORF3 and viral gRNA as a target could reflect that the viral RNA polymerase is able to make a continuous transcript, while achieving the similar cDNA synthesis in the RT-qPCR process is difficult.

We subsequently evaluated the relative amounts of mRNA transcripts containing ORF3 versus ORF2 in fish where the predominant form of viral RNA in the heart was identified as mRNA. Our findings revealed that, relative to ORF2-containing transcripts, transcripts containing ORF3 were present in significantly lower quantities in the heart compared to the kidney. The higher transcription activity but less efficient transcription of ORF3 could indicate a premature termination rather than the synthesis of full-length mRNA. Cytoplasmic core particles of dsRNA viruses typically synthesize full-length copies of the genome, which would lead to equal amounts of ORF3- and ORF2-containing transcripts. However, our results indicate a transcript termination before the end of ORF3. This finding could represent a regulatory mechanism employed by the virus to modulate the relative amounts of viral proteins or to generate various truncated forms of the ORF3 protein. Such a mechanism might enhance the virus protein variability.

Additionally, previous unpublished work referenced in an earlier publication from expression studies of PMCV ORF3 in cell cultures indicates the presence of truncated ORF3 forms [35].

We also pursued and searched for other possible natural hosts of PMCV beyond salmon. We used PCR and DNA extracted from organs of PMCV-positive fish as targets, using primers from conserved parts of the genome for various groups of organisms. These primers were originally developed for barcoding, i.e., taxonomic placement. Earlier phylogenetic studies have indicated that PMCV is most genetically related, among the classical totivirus genera, to the genus *Giardiavirus* [5,35], but also possibly to the totiviruses of arthropods [36]. Several of the totiviruses associated with fish are clustered to a separate genus tentatively called Pistolvirus. We tested quite broadly, with an emphasis on unicellular organisms. Using this approach, the only positive amplifications came from using primers from fungi, and sequencing of the amplicon showed a very high identity match to the fungi Malassezia spp. and Debaryomyces spp. However, relatively few PMCV-positive samples were positive for these fungi when tested by PCR. Moreover, these fungi are almost ubiquitous even in the marine environment [37]. NGS data were also generated from CMS-infected material in the present study and contigs were analyzed for the presence of fungal reads and contigs. Malassezia spp./M. restricta could again be identified. Although we found similar fungal reads in unrelated datasets produced more than a decade apart, contamination could not be ruled out as a source of this finding. A possible avenue for future research could involve screening items such as equipment, treatment nets, and biofilms for the presence of PMCV and its potential alternate hosts. Our findings of PMCV in skin mucus could indicate there may be an external source.

## 5. Conclusions

High viral PMCV RNA loads predominantly found in the spongiform layer of the heart are attributed to extensive viral mRNA transcription. There is a correlation between PMCV viral mRNA loads and the severity of histopathological changes observed through ISH techniques. This correlation suggests that the observed changes result from extensive viral transcription and its products, rather than cellular damage caused by the production of new viral particles. The production of full-length transcripts is regulated, with a reduction in the relative number of ORF3-containing transcripts at high transcription rates. The low or undetectable production of new viral particles in the heart, combined with extensive transcription activity and corresponding pathological consequences, suggests that the heart is partly a dead-end for virus production. Attempts to identify alternative hosts for PMCV, such as fungi, were inconclusive. These findings underscore the need for further research to fully understand PMCV’s life cycle and potential alternative hosts and its location when it is not infecting the hearts of Atlantic salmon.

## Figures and Tables

**Table 1:** Primers and probes used for the detection of viruses.

Virus	Primer/Probe	Sequence (5′ → 3′)	Amplicon (bp)	Reference
PMCV				
ORF2	PMCV-F	TTCCAAACAATTCGAGAAGCG	140	[6]
	PMCV-R	ACCTGCCATTTTCCCCTCTT		
	PMCV probe	FAM-CCGGGTAAAGTATTTGCGTC-MGBNFQ		
ORF3	Orf3-3′Fw	TTACAGAGGGCGGGAACCTGTGTGG		
	Orf3-3′Rw	TGGCTTCTTGTGAATTGTCAACAC	114	
	Orf3 probe	FAM-TCTTCGATAATACGCAGTGTA-MGBNFQ		
SYBR primers	SP3-orf2Fw	CTAAGGCCAGTGGCGGAATC	264	
	Orf2 Rw	TGGTGGCATACTTACCCATG		
	Orf3 Fw	GGCGAGAATGGTGTTTGTGCACTGC	266	
	SP3-orf3Rw	GAATGAAGCAAGATGGAACC		
	Orf2-3′Fw	TTGGGTTCAAGAGGATAGAG	122	
	Orf2-3′Rw	GAATTTTGGTACCTGTGATG		
PRV 1	Sigma3 659 Fw	TGCGTCCTGCGTATGGCACC		[18]
	Sigma3 801Rw	GGCTGGCATGCCCGAATAGCA		
	Sigma3_693 probe	FAM-ATCACAACGCCTACCT-MGBNFQ		
SAV	QnsP1 17F	CCGGCCCTGAACCAGTT		[19]
	QnsP1 122R	GTAGCCAAGTGGGAGAAAGCT		
	QnsP1_53probe	FAM-CTGGCCACCACTTCGA-MGBNFQ		

**Table 2:** Target and control probes for in situ hybridization.

Probe	Accession No.	Target Region (bp)	Catalog No.
V-piscine-myocarditis-ORF1-C2	JQ728724.1	1050–1757	812021-C2
V-PMCV-ORF2	HQ339954.1	3441–4500	555231
V-PMCV-ORF2-sense-C3	HQ339954.1	3441–4500	1219761-C3
DapB (negative control)	EF191515	414–862	310043
PPIB (positive control)	NM_001140870	20–934	494421

**Table 3:** Cq values measured by RT-qPCR for PMCV in various organs of a clinically healthy, PMCV-infected population of Atlantic salmon, two months after PMCV had been detected at the farm.

Fish No.	Heart	Gills	Kidney	Spleen	Liver	Muscle	Skin Scrape	Pyloric Ceca	Midgut	Hindgut
1	-	-	-	-	-	-	35.9	-	-	-
2	24.5	26.0	22.9	21.9	29.9	27.4	26.4	27.5	25.7	27.5
3	-	-	-	-	-	-	-	-	-	-
4	16.4	28.6	22.5	22.7	26.7	26.8	28.4	26.3	25.3	26.3
5	36.9	-	35.0	-	-	-	37.4	-	-	-
6	-	-	-	-	-	-	-	-	-	-
7	24.4	26.4	22.9	22.9	29.5	28.1	29.9	27.4	22.2	27.1
8	22.9	28.8	21.9	21.8	27.1	27.8	29.6	29.6	29.1	30.5
9	16.9	28.0	24.5	24.9	30.5	28.4	28.7	28.9	28.3	27.7
10	26.2	26.3	23.9	25.1	30.8	28.0	27.3	29.5	26.5	27.6
Ave	21.9	27.3	23.1	23.2	29.1	27.8	28.4	28.2	26.2	27.8

**Table 4:** Relative amounts of viral ssRNA and dsRNA in the heart and kidney.

	Heat-Treated Samples (ssRNA + dsRNA)		Non-Heat-Treated Samples (ssRNA)		ΔCq for PMCV ssRNA versus Total PMCV RNA	
Fish No.	Heart	Kidney	Heart	Kidney	Heart	Kidney
1	17.05	21.66	17.23	25.47	0.18	3.81
2	13.36	20.20	13.32	23.06	−0.04	2.86
3	14.19	20.26	14.49	24.24	0.3	3.94
4	12.79	21.74	12.78	25.27	−0.01	3.53
5	17.70	19.00	18.65	23.21	0.95	4.21
6	21.8	23.5	21.6	24.5	−0.2	1
7	17.7	20.6	17.6	24.2	−0.1	3.6
8	13.6	19.3	13.7	22.9	0.1	3.6
9	17.4	22.7	17.3	25.9	−0.1	3.2
10	17.5	20.4	17.6	24.5	0.1	4.1

**Table 5:** Relative amount of ORF3 ssRNA versus ORF2.

	ORF2	ORF3	ΔCq	Relative Amount of ORF3 ssRNA versus ORF2
Heart 2	12.08	15.36	−3.28	10%
Heart 3	12.97	17.94	−4.97	3%
Heart 4	11.34	16.60	−5.26	3%
Heart 5	18.31	21.72	−3.41	9%
**Average**	13.675	17.90		6.25%
Kidney 2	22.91	24.23	−1.32	40%
Kidney 3	23.84	25.43	−1.59	33%
Kidney 4	24.68	27.10	−2.42	19%
Kidney 5	23.13	24.71	−1.58	33%
**Average**	23.64	25.37		31.25%

## Data Availability

The raw data supporting the conclusions of this article will be made available by the authors without undue reservation.

## Supplementary Materials

The following supporting information can be downloaded at https://www.mdpi.com/article/10.3390/pathogens13090744/s1, Figure S1: In situ hybridization of the heart ventricle with fluorescent probes detecting PMCV ORF1 and ORF2, wherein the same section was used and the probes detected the same cells. Table S1: Primers used for different classes of organisms [38,39,40,41,42,43,44,45,46,47,48].
