# Supplementary material for: Evaluating Atlantic Salmon (Salmo salar) as a Natural or Alternative Host for Piscine Myocarditis Virus (PMCV) Infection
Source: Pathogens. 2024 Aug 30;13(9):744. doi: 10.3390/pathogens13090744 (PMC11434702; doi:10.3390/pathogens13090744)
Supplement: Supplementary file 1 [file pathogens-13-00744-s001.zip › pathogens-3115533-supplementary.pdf]

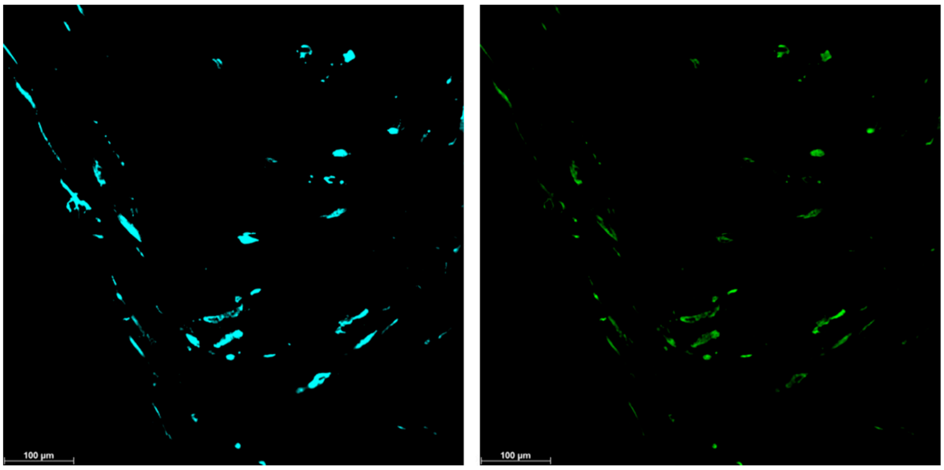

Figure S1 In situ hybridization of heart ventricle with fluorescent probes detecting PMCV ORF1 and ORF2. Same section. The probes detect the same cells.

Table S1. Primers used for different classes of organisms [38–48].

Target	Primer name	Sequence 5′- 3′	Reference
**Fungi**	ITS1	TCCGTAGGTGAACCTGCGG	[38]
	ITS4 (ytre)	TCCTCCGCTTATTGATATGC	
**Fungi**	ITS2	GCTGCGTTCTTCCATCGATGC	[38]
	ITS3	GCATCGATGAAGAACGCAGC	
**Fungi**	NL-1	GCATATCAATAAGCGGAGGAAAAG	[38]
	NL-4	GGTCCGTGTTTCAAGACGG	
**Fungi**	EF1-1018F	GAYTTCATCAAGAACATGAT	[38]
	EF1-1620R	GACGTTGAADCCRACRTTGTC	
**Fungi**	ITS5	GGAAGTAAAAGTCGTAACAAGG	[39]
	ITS4	TCCTCCGCTTATTGATATGC	
**Trypanozoma**	Try-Fw	CCAWACAACAAACATATGATGCTGC	[40]
	Try-Rw	TCCHGATATGGTWTTKCCYCG	
**Apicomplexa**	Api-Fw	GAAACTGCGAATGGCTCATT	[41]
	Api-Rw	CTTGCGCCTACTAGGCATTC	
**Mikrosporidia**	CM-V5F	GATTAGANACCNNNGTAGTTC	[42]
	CM-V5R	TAANCAGCACAMTCCACTC	
**Mikrosporidia**	bcdF01	CATTTTCHACTAAYCATAARGATATTGG	[42]
	bcdR06	GGDGGRTAHACAGTYCAHCCNGT	
**Myxozoa**	Myxo_617F	CGCGCAAATTACCCAMTCCA	[43]
	Myxo_2313R_all	CGTTACCGGAATRRCCTGACAG	
**Ichtyophonus**	NS1-F	GTAGTCATATGCTTGTCTC	[44]
	NS8 deg-R	TCCGCAGGTTCACCWACGGA	
**Ichtyophonus**	vc7F	GTCTGTACTGGTACGGCAGTTTC	[44]
	vc5R	TCCCGAACTCAGTAGACACTCAA	

**Ichthyophonus**	Out ITS1-F<br>Out-ITS2-R	GCGGAAGGATCATTACCAAATAACG<br>GCCTGAGTTGAGGTCAAATTT	[44]
**Ichthyophonus**	Ich7f<br>Ich6r	GCTCTTAATTGAGTGTCTAC<br>CATAAGGTGCTAATGGTGTC	[44]
**Malassezia**	LROR<br>LR5	ACCCGCTGAACTTAAGC<br>TCCTGAGGGAAACTTCG	[45]
**Malassezia**	Mal63 Fw Mal487 Rw	TTGGCTACAGCGGCGACGACCTG<br>CATCGCCTTGCCGACCGTCG	[45]
**Malassezia**	LR5 - 2<br>LROR-2	ATCCTGAGGGAAACTTC<br>GTACCCGCTGAACTTAAGC	[45]
**Malassezia**	Mala_28S_F2<br>Mala_28S_R2	CGCGTTGTAATCTCGAGACG<br>CCACCCAAAAACTCGCACA	[45]
**Debaryomyces**	Deb Fw<br>Deb Rw	TCACCATCTTTCGGGTCCCAACAGC<br>CCCGTGCGATGAGATGCCCAATTC	[46]
**Oomycetea**	Cox2-F<br>Cox2-R	GGCAAATGGGTTTTCAAGATCC<br>CCATGATTAATACCACAAATTTCACTAC	[47]
**Crusteacea**	LCO1490<br>HCO2198	GGTCAACAAATCATAAAGATATTGG<br>TAAACTTCAGGGTGACCAAAAAATCA	[48]
